# Dysbiosis of the Vaginal Microbiota and Higher Vaginal Kynurenine/Tryptophan Ratio Reveals an Association with *Chlamydia trachomatis* Genital Infections

**DOI:** 10.3389/fcimb.2018.00001

**Published:** 2018-01-18

**Authors:** Noa Ziklo, Miranda E. Vidgen, Kuong Taing, Wilhelmina M. Huston, Peter Timms

**Affiliations:** ^1^Faculty of Science, Health, Education and Engineering, University of the Sunshine Coast, Sippy Downs, QLD, Australia; ^2^Sexual Health and HIV Service, Clinic 87, Sunshine Coast, Nambour, QLD, Australia; ^3^School of Life Sciences, Higher Education and Academic Practice, University of Technology, Sydney, NSW, Australia

**Keywords:** tryptophan, kynurenine, indole, vaginal microbiota, interferon-gamma, *Chlamydia trachomatis*

## Abstract

The natural course of *Chlamydia trachomatis* urogenital tract infections varies between individuals. While protective immunity can occur, some women can become reinfected, contributing to the development of severe pathology. While the reasons for these differences are unknown, an individual's response to induced interferon-γ (IFN-γ) is suggested to be critical. IFN-γ induction of the enzyme indoleamine 2,3-dioxygenase, which depletes tryptophan, may be the key. One hypothesis suggests that indole-producing bacteria in the vaginal microbiota can provide a substrate for the *Chlamydia* to synthesize tryptophan, rescuing the *Chlamydia* from host IFN-γ attack. We studied a cohort of 25 women who were either, *Chlamydia* negative, *Chlamydia* positive with a single infection, or *Chlamydia* positive with repeated infection, to test our hypothesis. We characterized their vaginal microbiota, cytokine response, as well as their tryptophan, kynurenine and indole concentrations directly in vaginal secretions. We found that *C. trachomatis* urogenital tract infections either initial or repeat infections, were associated with elevated vaginal kynurenine/tryptophan ratios, primarily as a result of elevated kynurenine levels. In addition, vaginal microbiota of community state type (CST) IV showed significantly lower vaginal tryptophan levels compared to CST I and III, which might be related to a higher abundance of indole producers found within this group. Furthermore, we found a higher abundance of indole producers in women who cleared their *Chlamydia* infection post antibiotic treatment. This study demonstrates for the first time *in vivo*, the association between high vaginal kynurenine/tryptophan ratios and *C. trachomatis* infections. In addition, tryptophan depletion was associated with vaginal microbiota of CST IV.

## Introduction

Genital infections with *Chlamydia trachomatis* continue to be highly prevalent despite sensitive diagnosis and effective antibiotic treatments being available (Lanjouw et al., 2015; Newman et al., 2015). Despite all efforts, the natural course of *C.trachomatis* infections are still not fully understood nor is the precise immune profile of protection. While some women apparently do develop some level of protective immunity (Brunham et al., 1996; Cohen et al., 2005; Batteiger et al., 2010), others are susceptible to repeated infections that can result in severe pathology. One of the most important immune responses for the development of protective immunity against *Chlamydia* infection is characterized by antigen-specific interferon-gamma (IFN-γ) secretion by CD4+ T cells (Cohen et al., 2000, 2005; Barral et al., 2014; Hafner et al., 2014). IFN-γ is considered to be an anti-chlamydial agent due to its ability to upregulate the enzyme indoleamine 2,3-dioxygenase (IDO1) (Byrne et al., 1986). IDO1 catabolizes the amino acid tryptophan into N-formylkynurenine (Takao et al., 1978), depleting the host cell tryptophan pools. *Chlamydia* is a tryptophan auxotroph and depends on the host pools of tryptophan to establish and maintain its infection (Mcclarty, 1994; Xie et al., 2002). However, unlike other *Chlamydia* sub-species (such as ocular trachoma strains), genital *C. trachomatis* strains are able to use exogenous indole which they can convert back to tryptophan using their functional tryptophan synthase gene (*trpBA*) (Caldwell et al., 2003). One hypothesis suggests that indole-producing bacteria in the vaginal microbiota might play a role in *C. trachomatis* infections by providing exogenous indole, allowing the *Chlamydia* to overcome the host IFN-γ-mediated immune response (Morrison, 2003). Indole producing bacteria may be prevalent in the female genital tract of some women, most likely with more diverse anaerobic vaginal bacterial communities and hence, this could be a direct source of indole / tryptophan for the *Chlamydia* (Ziklo et al., 2016a). Indole is an important signaling molecule produced by many bacterial species colonizing some of the mucosal sites in the human body, in particular the gastrointestinal tract (Lee and Lee, 2010). In addition, indole was previously detected in genital secretions of two women who were infected with *Chlamydia* (Lewis et al., 2014).

IDO1 can be produced by many cell types in response to specific cytokines such as IFN-γ, IL-1β, TNF-α, and IL6 (Shirey et al., 2006; Campbell et al., 2014). IDO1 production has been associated with reduced differentiation of IL-17 producing CD4+ T cells (Favre et al., 2010), increased production of anti-inflammatory IL-10 (De Luca et al., 2013), tolerance and immunosuppression (Moffett and Namboodiri, 2003). Catabolism of tryptophan through the kynurenine pathway has also been shown to be involved in many diseases and disorders (Campbell et al., 2014; Heng et al., 2016), while kynurenine specifically has been shown to be a very important biomarker for disease progression (Chen and Guillemin, 2009), and has been associated with dysbiosis of the gut microbiota (Vujkovic-Cvijin et al., 2014). However, as an immune-suppressor, IDO1 has been found to have beneficial effects in tolerance and one study reported that high kynurenine was associated with a healthy state of the vaginal microbiome in comparison to women with bacterial vaginosis (BV) (Vitali et al., 2015).

We know that the vaginal tract microbiota has an important role in health and disease and might have severe consequences for reproductive outcomes (White et al., 2011; Moreno et al., 2016), as well as reproductive disorders, such as pelvic inflammatory disease, endometriosis, ectopic pregnancy, preterm birth and tubal factor infertility (McGregor, 2000; Oakeshott et al., 2002; Ness et al., 2005; Durugbo et al., 2015). BV is a state in which the vaginal microbiome shifts from being dominated by key *Lactobacillus* spp. to one with a higher proportion of non-lactobacilli communities. BV is also characterized by higher vaginal pH levels, fishy odor and discharge and has been shown to be highly associated with sexually transmitted infections such as *C. trachomatis*, as well as reproductive complications (Wiesenfeld et al., 2003; Brotman et al., 2010; White et al., 2011). Community state types (CST) are used to characterize vaginal microbiota based on the dominant *Lactobacillus* species (CST I, II, III, and IV) (Ravel et al., 2011). However, CST IV is characterized by a higher abundance of anaerobic non-*Lactobacillus* bacteria, such as *Prevotella, Atopobium, Gardnerella, Parvimonas, Megasphaera, Peptoniphilus*, and *Streptococcus*, and is often associated with higher pH and higher Nugent score (Ravel et al., 2011; Huang et al., 2014; Albert et al., 2015). The relationship between the vaginal microbiota and its host are orchestrated by important biochemical compounds, among them are tryptophan and kynurenine produced either by the host's catabolic pathways, the microbiome or mycobiome colonizing the area and often have an effect on the host immune response (De Luca et al., 2013; Mirmonsef et al., 2014; Romani et al., 2014; Nelson et al., 2015).

This study aimed to answer the question evolving around a decade-old hypothesis, in regards to the indole-producing bacterial proportion within the genital microbiome that potentially contributes to *C. trachomatis* immune evasion through tryptophan starvation. We have previously found that indole-producing bacterial supernatant was able to rescue *C. trachomatis* after tryptophan depletion *in vitro* (Ziklo et al., 2016b). We have expanded this *in vitro* data to analyze a small cohort of women attending a Sexual Health Clinic, either *Chlamydia* negative, *Chlamydia* positive or *Chlamydia* repeat-positive. We measured the women's microbiome, cytokine production, along with vaginal tryptophan, kynurenine and indole levels. In this study, we provide evidence that women with repeated *C. trachomatis* genital infection have significantly higher kynurenine and kynurenine/tryptophan ratio levels, while women with CST IV have significantly low levels of tryptophan.

## Materials and methods

### Study cohort

We have collected 37 samples from 25 women, who attended the Sexual Health Clinic in Nambour, Australia, at different time points (Supplementary Table 1). Women were diagnosed for Chlamydia infection by the clinic using Cobas® 4800 CT/NG Test (Roshe, Australia) (Rockett et al., 2010), and were either *Chlamydia trachomatis* negative (CT-N; *n* = 10), Chlamydia positive with first infection (CT-P; *n* = 11) or Chlamydia positive with repeated infection in the past year (CT-RP; *n* = 3). Women with first current Chlamydia infection were treated with azithromycin 500 mg and were invited to second and third visits for follow up [post antibiotic treatment (PAT); *n* = 13]. Two high vaginal swab samples, one cervical secretion sample and a 5 ml blood sample were collected from each woman. Women's samples were diagnosed for the presence of clue cells using Gram stain along with vaginal pH and Nugent score. This study was carried out in accordance with the recommendations of University of the Sunshine Coast, Human Ethics Committee (number A/14/623), and Prince Charles Hospital Human Research Ethics (number HREC/14/QPCH/14), with written informed consent from all subjects. All subjects gave written informed consent in accordance with the Declaration of Helsinki. The protocol was approved by the University of the Sunshine Coast, Human Ethics Committee and Prince Charles Hospital Human Research Ethics.

### Elution of vaginal secretions

LASIK PVA eye sponges (Visitec, USA) were placed in the posterior fornix of the vagina for 2 min to absorb secretions. Sponges were immediately placed at −20°C until vaginal fluids were eluted at the same day, as previously described by Kozlowski et al. (2000), using 300 μl of PBS containing 1% Bacterial Protease Inhibitor (Sigma, Australia) and 10% igepal (Sigma, Australia).

### Tryptophan and kynurenine ELISA

Tryptophan and kynurenine concentrations in vaginal secretions samples were determined using tryptophan and kynurenine ELISA assay (Immusmol, France), according to the manufacturer's instructions. Briefly, 20 μl of cervical secretion was used from each sample in a 96-well plate competitive ELISA assay. Measurements of O.D. were converted into concentrations according to a standard curve. Measurements were corrected according to each of the samples' dilution factor. Kynurenine/tryptophan ratio was calculated by dividing the measurements from the two kits.

### Measurement of cytokines in vaginal secretions

Three cytokines, IFN-γ, IL-17, and IL-10 were measured from women's cervical secretions, using ELISA assay (Elisakit.com, VIC, Australia), according to the manufacturer's instructions. Protein levels in pg/ml were determined according to a standard curve and measurements were corrected according to each of the samples' dilution factor.

### Analysis of the vaginal microbiome

High vaginal swab samples were stored in 1 ml of RNA*later* at −80°C until processed. DNA was extracted from the swabs using QIAamp Mini Kit (Qiagen, Australia) according to the manufacturer's instructions with minor changes. Briefly, swab tips stored in RNA*later* were vortexed for 3 min, then 0.5 ml were transferred into a new 1.5 ml sterile Eppendorf tube and centrifuged for 30 min at 16,000 × g. Supernatant was discarded and pellet was resuspended in 50 μl of sterile Tris-EDTA buffer (TE, pH 8). Samples were heated to 95°C for 10 min, cooled, then 130 μl of ATL buffer and 20 μl of proteinase K were added. Samples were then incubated overnight at 56°C. Following incubation, extraction method was performed per manufacturer's kit instructions. DNA concentrations and purity were determined using Qubit (Thermo-Fisher, Australia). PCR amplification of the V3-V4 hypervariable regions of the 16S rRNA gene was performed using dual-indexed primers (Fadrosh et al., 2014). PCR amplicons of the samples and controls were created using barcoded 319F and 806R primers at a concentration of 1 μM with HiFi Hot Start Ready Mix (KAPA Biosystems, Cape Town, South Africa). The reaction was performed on a Nexus Mastercycler (Eppendorf, Hamburg, Germany) with 3 min of initial denaturation at 95°C, followed by 30 cycles at 95°C for 15 s, 55°C for 15 s, and 72°C for 30 s and a final extension step at 72°C for 5 min. PCR products were cleaned and standardized using SequalPrep™ Normalization Kit (Invitrogen, Carlsbad, CA) and then pooled. Library was then concentrated using DNA Clean and Concentrator-25 (Zymo Research, Irvine, CA), and primers removed using a Gel DNA Recovery Kit (Zymo Research; Irvine, CA). The libraries were sequenced on the MiSeq platform using 600-cycle kit chemistry v3 (Illumina, San Diego, CA). The raw MiSeq data files were pre-processed using the method previously described (Fadrosh et al., 2014), with PEAR for merging the paired end reads (Zhang et al., 2014), CutAdapt for trimming barcode/primer (Martin, 2011), and vsearch for detection and removal of chimeric sequences (Rognes et al., 2014). Pre-processed sequence data were then analyzed using QIIME (v1.9) (Caporaso et al., 2012). Operational taxonomic units (OTUs) were picked using a closed-reference strategy (Rideout et al., 2014) using a previously published vaginal specific 16S database (Srinivasan et al., 2012), which was modified to contain additional indole producing bacteria known to be present in the vaginal microbiome (Ziklo et al., 2016a).

### Statistical analysis

Data analysis was performed in Graph Pad Prism V. 7.01 (Graph Pad Software, Australia) and presented as mean ± standard deviation (SD). Statistical differences were analyzed using Mann-Whitney test. Correlation analysis was performed using Pearson test. Comparative analysis of specific bacteria in the vaginal microbiome data was performed using Kruskal Wallis test in QIIME (Caporaso et al., 2012). Contingency tables were analyzed using Fisher's exact test.

## Results

### Higher kynurenine/tryptophan ratio levels are present in *Chlamydia* positive (single or repeat infections) compared to *Chlamydia* negative women

**Figure 1:**
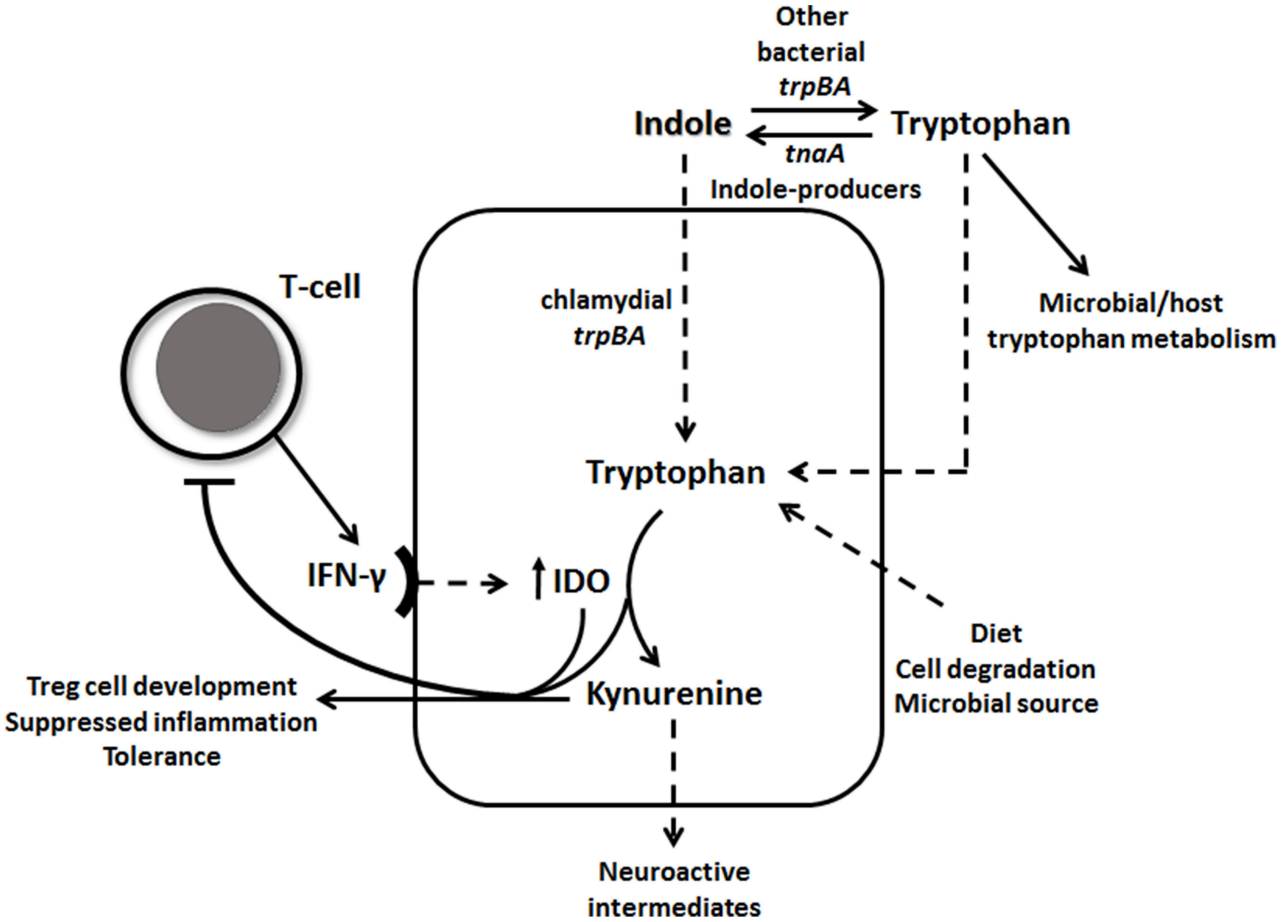
IFN-γ-tryptophan-kynurenine and indole pathways. IFN-γ induces IDO1 levels, which catabolizes tryptophan through the kynurenine pathway. Tryptophan depletion and high levels of kynurenine down-regulates IFN-γ activity. Chlamydial tryptophan synthase gene (*trpBA*) converts available indole back to tryptophan, as well as other vaginal tract bacterial *trpBA*. Indole-producing bacteria can synthesis indole from tryptophan using *tnaA* gene. Tryptophan can be accumulated in the epithelial cell in many ways, such as through diet, cell degradation and microbial source.

**Figure 2:**
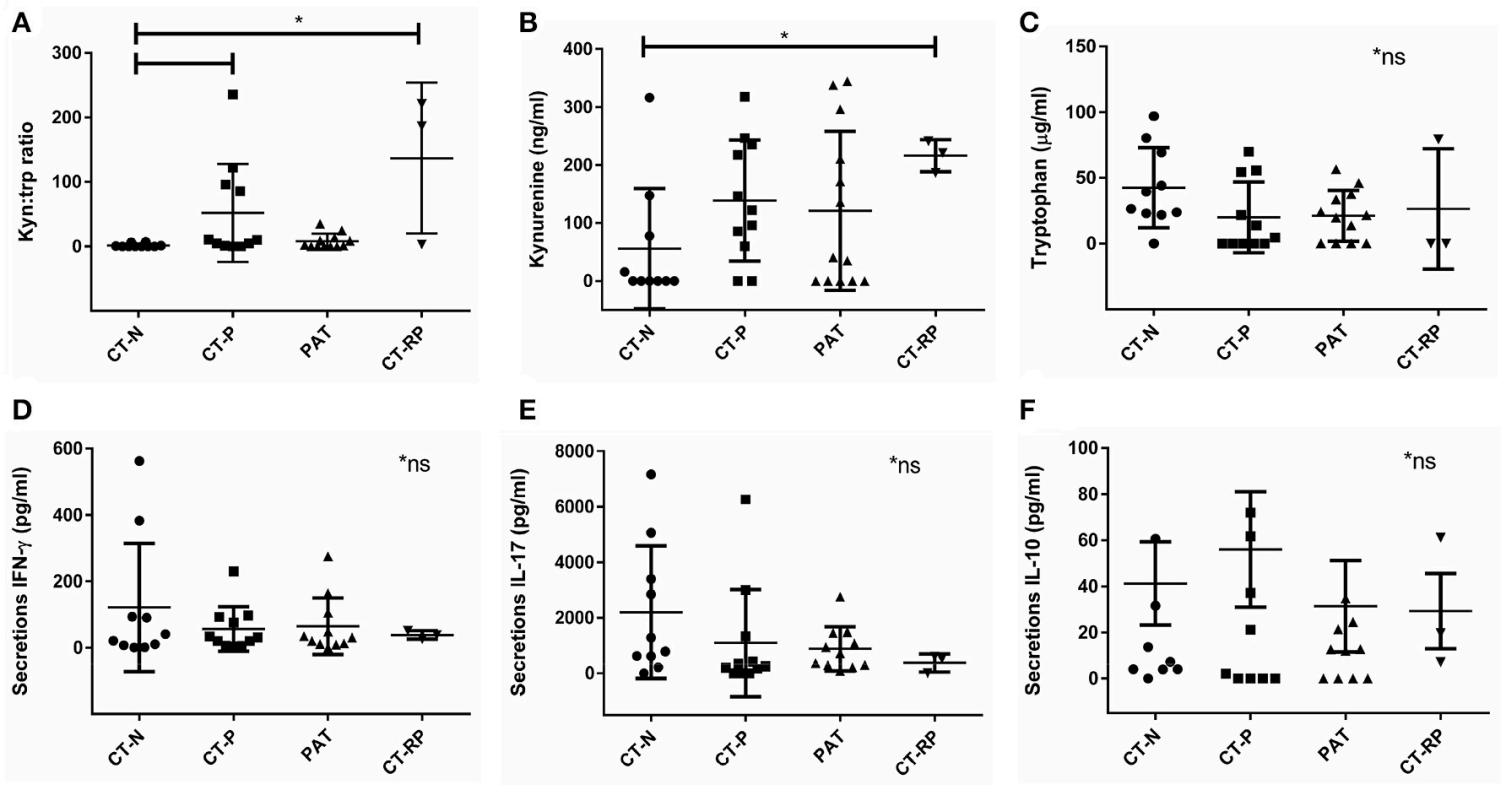
Vaginal kynurenine and tryptophan levels according to women's chlamydial infection status and their association with vaginal cytokine response. **(A)** Kynurenine/tryptophan ratio, **(B)** kynurenine (ng/ml), and **(C)** tryptophan (μg/ml) comparison between *Chlamydia* negative (CT-N; *n* = 10), *Chlamydia* positive (CT-P; *n* = 11), post antibiotic treatment (PAT; *n* = 13) and repeated *Chlamydia* infections (CT-RP; *n* = 3). Cytokines **(D)** IFN-γ, **(E)** IL-17, and **(F)** IL-10 levels (pg/ml) measured from women's vaginal secretions samples according to their *Chlamydia* infection status. Significant differences are indicated in the figure, using Mann-Whitney test, *p* < 0.05. Non-significant results are indicated in the figure (^*^ns). Data are presented as mean with SD.

IFN-γ is known to be a key regulator of chlamydial growth and one of the mechanisms is via IDO1 upregulation and depletion of tryptophan, by conversion into kynurenine (Figure 1). To determine if vaginal tract levels of available tryptophan or its product, kynurenine, might correlate with an individual's response to initial or repeat infections with *C. trachomatis*, we measured protein levels of tryptophan and kynurenine from vaginal secretion. While we did not find a significant correlation with tryptophan levels alone (Figure 2C), we did find that *Chlamydia* positive women, with either single or repeated infection, had significantly higher kynurenine/tryptophan ratios (*p* < 0.005) when compared to *Chlamydia* negative women (Figure 2A). In addition, women with repeated *Chlamydia* infections had significantly higher kynurenine levels in comparison to *Chlamydia* negative women (*p* < 0.05, Figure 2B).

### Women with repeated chlamydial infections have reduced levels of IFN-γ and IL-17

Higher kynurenine/tryptophan ratio levels in women with an active *Chlamydia* infection supports the hypothesis of an anti-chlamydial IFN-γ immune response to the pathogen, subsequently, activating the enzyme IDO1 to deplete tryptophan into kynurenine. However, when looking at the vaginal secretion IFN-γ response of the women in our cohort, although not significantly different, we did find that women with repeated chlamydial infections (CT-RP) actually had reduced levels of IFN-γ, as well as IL-17 (Figures 2D,E), in comparison with *Chlamydia* negative women. In addition, we found that women with a single chlamydial infection (CT-P) had slightly higher vaginal IL-10 levels, although not significantly different (Figure 2F).

### Women with CST IV have the lowest vaginal tryptophan levels

**Figure 3:**
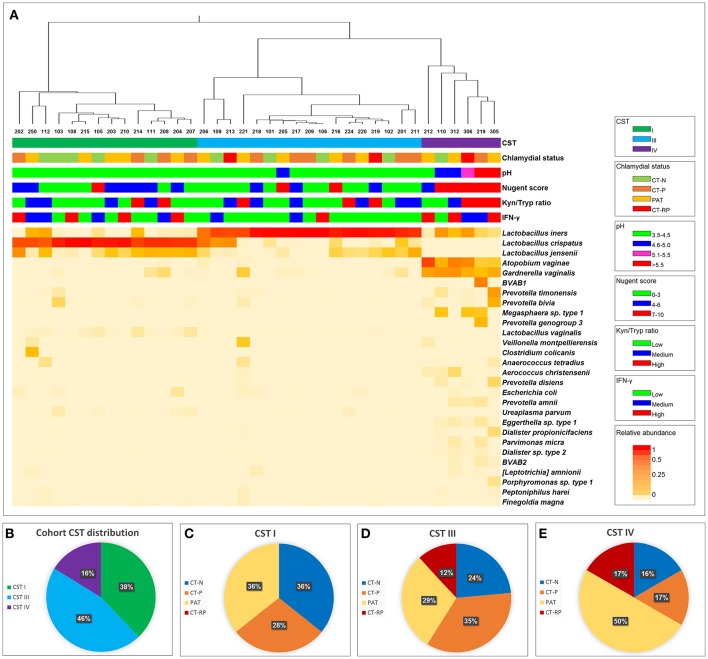
Vaginal taxonomic distribution. **(A)** Heatmap of vaginal microbial taxa detected in women who were either *C. trachomatis* negative (chlamydial status: CT-N in green), *C. trachomatis* positive single infection (CT-P in orange), samples were taken from women with current infection at second and third visit, post antibiotic treatment (PAT in yellow) and *C. trachomatis* positive with repeated infection in the past year (CT-RP in red). Relative abundance of each taxa (species level where possible) is presented by color, with red indicating higher abundance, and lighter shades of orange indicating lower abundance. To the right of the heatmap is the nearest species as assigned by modified vaginal specific 16S database (Srinivasan et al., 2012), with the addition of several indole producing bacteria known to be found in the human vaginal microbiome. For each of the women's sample, CST, chlamydial status, pH, Nugent score, kynurenine/tryptophan ratios and vaginal IFN-γ response data are presented. **(B)** Taxonomic distribution of CST I, III, and IV in our overall cohort. **(C)** Women's chlamydial infection status distribution (CT-N, CT-P, PAT, and CT-RP), among CST I, **(D)** CST III and **(E)** CST IV, presented as percentages.

The metabolic pathway involving IFN-γ-induced tryptophan depletion into kynurenine is regulated by many factors that might induce or inhibit IDO1 activity. One key source of regulation might be the vaginal microbiome, with healthy women having a microbiome dominated by lactobacilli and in comparison, women with dysbiosis (such as CST IV dominant and BV) having fewer lactobacilli and more anaerobic bacteria. We determined the vaginal 16SrRNA microbiota profiles for all women in our trial (Figure 3). In our cohort, 38% of the women had a microbiome of CST I, 46% of the women had CST III and 16% had CST IV (Figure 3B). Women with CST IV had a higher Nugent score of 7-10 (Fisher's exact test, *p* = 0.0007), pH ≥ 5 (*p* < 0.0001) (Figure 3A) and higher presence of clue cells according to Gram stain (*p* = 0.0197) (not shown). Women with CST IV (16%) were less likely to be *Chlamydia* negative than those with CSTI (36%) or CSTIII (24%) (Figures 3C–E). Although this is not statistically significant (Fisher's *p* = 0.367), this trend suggests an association between previous or current chlamydial infections and vaginal microbiota dysbiosis as characterized by CST IV.

**Figure 4:**
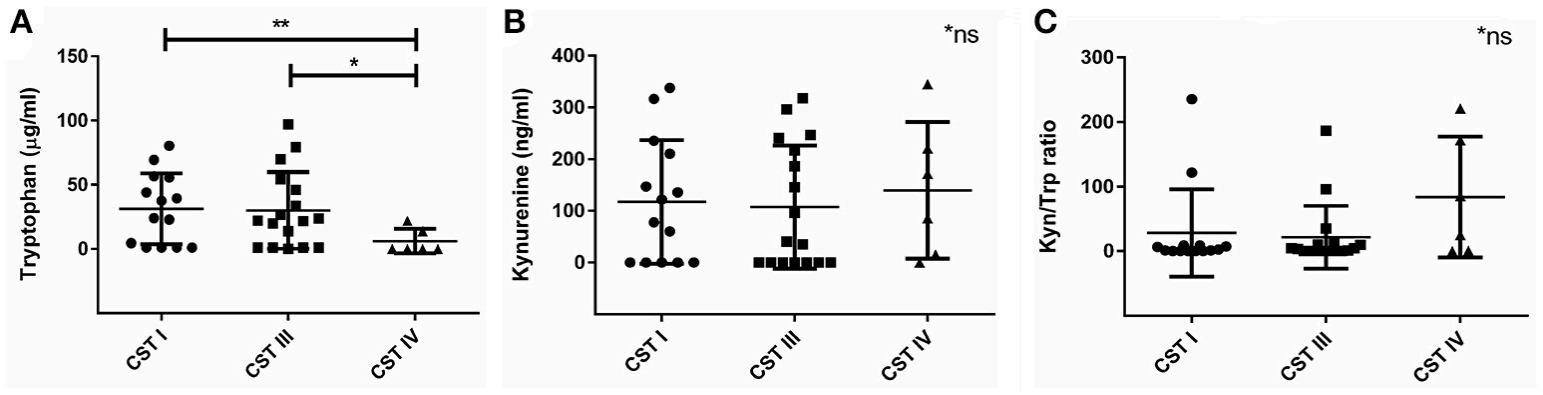
Vaginal tryptophan and kynurenine levels in women according to their CST. **(A)** Tryptophan (μg/ml), **(B)** kynurenine (ng/ml), and **(C)** Kynurenine/tryptophan ratio, in women with CST I (*n* = 14), III (*n* = 17) and IV (*n* = 6). Women with CST IV had significantly lower levels of tryptophan in compare to women with CST I (^**^p = 0.0058) and III (^*^p = 0.0126), using Mann-Whitney test. Data are presented as mean ± SD.

Next, we wanted to investigate the association we found between vaginal microbial community composition and the biochemical factors associated with *C. trachomatis* infections. Lactic acid is known to inactivate *C. trachomatis* (Gong et al., 2014), therefore, the absence of *Lactobacillus* spp. in women with CST IV might promote a suitable environment for the *Chlamydia*. Apart from lactic acid, kynurenine and tryptophan levels are also expected to have an impact on chlamydial infection. Despite the relatively low sample size, we found that women with CST IV had significantly lower tryptophan levels in comparison to women with CST I (*p* = 0.0058) and women with CST III (*p* = 0.0126) (Figure 4A). On the other hand, this time kynurenine levels were the same in women with CST I, III, and IV (Figure 4B) suggesting that it is unlikely that tryptophan depletion is due to host cell IDO1 activity (Figure 4C). Perhaps this suggests that low tryptophan levels are due to metabolism of some of the members in the bacterial community of CST IV and are related to dysbiosis. In addition, when evaluating the tryptophan and kynurenine levels according to *Chlamydia* infection status in each CST, we found that for women who are *Chlamydia* negative, CST is a strong predictor for tryptophan levels. This was indicated by the observation that women with CST I had higher tryptophan levels, followed by CST III and CST IV with lowest levels (Supplementary Figure S1A). In addition, CST IV and repeated *Chlamydia* infection are strong estimators for high kynurenine levels as well as high kynurenine/tryptophan ratios (Supplementary Figures S1B,C).

### Regardless of CST classification, higher abundance of *Streptococcus* spp. and *Peptoniphilus* spp. were found in women with higher kynurenine/tryptophan ratios

**Figure 5:**
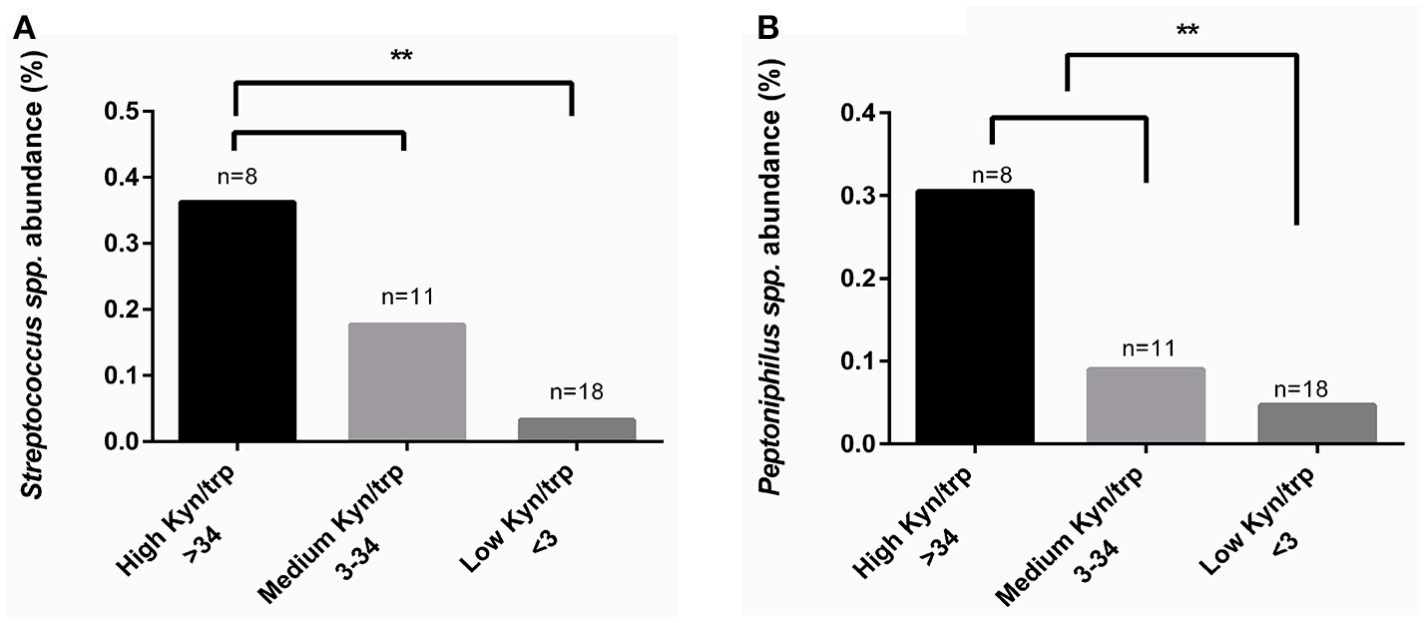
Comparative analysis of kynurenine/tryptophan ratio measured from the women's secretions samples with the mean relative abundance of OTUs summarized to genus level. **(A)** *Streptococcus* spp. and **(B)** *Peptoniphilus* spp., higher abundance correlated with high kynurenine/tryptophan ratio. The kynurenine/tryptophan ratio values are “low” < 3, “medium” 3 < 34 and “high” > 34. Statistical differences are indicated in the graph (^**^p < 0.05), using Kruskal Wallis test.

Because we could not find any differences in vaginal kynurenine levels of women with CST I, III, and IV, we hypothesized that the low tryptophan levels might be due to tryptophan metabolism in some of the bacterial members of CST IV. We conducted a comparative analysis with kynurenine, tryptophan and kynurenine/tryptophan ratio levels using Kruskal-Wallis test. Although we could not find a significant correlation with the individual measurements of kynurenine and tryptophan (not shown), we did find that a high kynurenine/tryptophan ratio significantly correlated with higher abundance of *Streptococcus* spp. (*p* = 0.017) and *Peptoniphilus* spp. (*p* = 0.039) (Figures 5A,B). This might suggest that there are specific bacteria, commonly found in CST IV although occasionally in other CSTs, that are responsible for high kynurenine/tryptophan ratio levels, either due to elevated immune response or through an active tryptophan metabolism.

### Indole-producing bacteria were found in 94% of the samples

**Figure 6:**
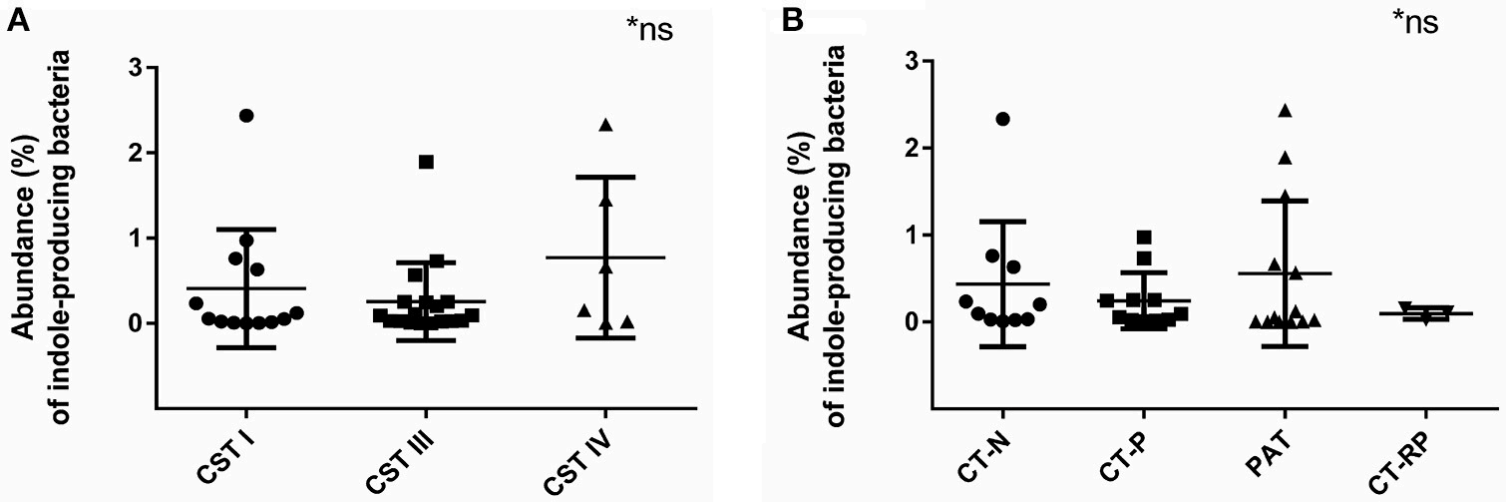
Relative abundance of indole-producing bacteria according to women's CST and their *Chlamydia* infection status. **(A)** Abundance of indole producing bacteria in women with CST I (*n* = 14), III (*n* = 17), and IV (*n* = 6). **(B)** Abundance of indole-producing bacteria in women who were *Chlamydia* negative (CT-N; *n* = 10), *Chlamydia* positive (CT-P; *n* = 11), post antibiotic treatment (PAT; *n* = 13) and repeated *Chlamydia* infections (CT-RP; *n* = 3). No significant differences were found. Data are presented as mean ± SD.

Indole-producing bacterial species *Porphyromonas asaccharolytica, Propionibacterium acnes, Fusobacterium nucleatum, Faecalibacterium prausnitzii, Enterococcus faecalis, Peptoniphilus harei*, and *Escherichia coli*, were found in 94% of the samples analyzed, with maximum relative abundance among individuals being 2.4% of reads. Higher relative abundance of indole-producing bacteria were found among women with CST IV (Figure 6A), and in women PAT (although not statistically significant) (Figure 6B). These results verify that indole-producing bacteria are commonly occurring within the female vaginal microbiota, and that microbiota dysbiosis and azithromycin treatment may be relevant to higher abundance of these bacterial species.

## Discussion

Our study provides the first *in vivo* evidence that the IFN-γ-Tryptophan-Indole-Microbiota axis is active during chlamydial infections and the composition of the microbiota in the genital tract may interplay considerably with the effectiveness of the host response to chlamydial infection.

A well established, and *in vitro* validated hypothesis in the field (Morrison, 2003), is that *C. trachomatis* infection triggers a host IFN-γ response which leads to the upregulation of IDO1, subsequently depleting tryptophan to kynurenine. Because genital *C. trachomatis* is a tryptophan auxotroph, for it to survive and cause reproductive infection, it must obtain tryptophan somehow, potentially via indole from indole-producing bacteria in the vaginal microbiota. We found that women with repeated *C. trachomatis* infections had significantly elevated vaginal kynurenine/tryptophan ratios. Despite the low number of participants, our results suggest that indeed the chlamydial infection did trigger an IFN-γ response and subsequent IDO1 production, which degraded pools of vaginal tryptophan to kynurenine. Interestingly, when we measured vaginal IFN-γ levels in these women, they actually had lower levels than in controls. This might not be surprising, as high kynurenine, along with low tryptophan levels were previously shown to alter T-helper cells and down-regulate/alter the immune response (including down regulation of IFN-γ) (Favre et al., 2010; Zelante et al., 2014). For example, *Chlamydia* positive participant 214, who had a high kynurenine/tryptophan ratio (235.6), with no vaginal tryptophan detected, had only low levels of IFN-γ (20.17 pg/ml). In addition, IL-17 levels were not detected from this individual, which supports the suggestion that low tryptophan and high kynurenine levels may suppress Th1 and Th17 immune response (Munn et al., 1999; Favre et al., 2010).

Dysbiosis of the vaginal microbiota is often characterized by elevated pH levels, Nugent score (Ravel et al., 2011), fishy odor (Nelson et al., 2015), discharge and diverse non-lactobacilli bacterial communities (Srinivasan et al., 2012; Ravel et al., 2013), which has been shown to alter the host immune response (Mitchell and Marrazzo, 2014; Mackelprang et al., 2015) and increase susceptibility to sexually transmitted infections and other opportunistic pathogens (Martin et al., 1999; Wiesenfeld et al., 2003). Consistent with this previous literature we found that women with CST IV had higher pH levels, Nugent score and higher presence of clue cells. Women with CST IV also had significantly lower vaginal tryptophan levels, but not higher kynurenine levels. This supports the proposal that the lower tryptophan levels in the women who had a CST IV microbiota was most likely due to tryptophan metabolism by the microbiota, and not IDO1 induced immune response. Furthermore, we also found that higher abundance of *Streptococcus* spp. and *Peptoniphilus* spp. (previously described to be associated with BV), was correlated with higher vaginal kynurenine/tryptophan ratio levels. This might indicate that vaginal kynurenine and tryptophan are not influenced solely by direct host IFN-γ-mediated immune response, but also via the presence of certain bacteria colonizing the genital tract, again, either due to modification of the immune response or by active tryptophan metabolism. Of the three *Peptoniphilus* spp. detected in the vaginal microbiota of women in our cohort, two of them, *P. duerdenii* and *P. harei* are tryptophan auxotrophs (Uniprot.org) and therefore, are predicted to exploit the host tryptophan pools, which may lead to low tryptophan levels. In addition, *P. harei*, which was found to have relatively high abundance (indicated in Figure 3), is an indole-producer, which may further deplete the host tryptophan pools. This suggests that the presence of *Peptoniphilus* spp. may be related to high kynurenine/tryptophan ratios due to active tryptophan metabolism by bacteria from this genus. Although from all seven different *Streptococcus* spp. detected in the vaginal microbiota of women in our study, only *Streptococcus agalactiae* is a tryptophan auxotroph (Uniprot.org), there is evidence that toxins being produced by *Streptococcus* spp. may influence tryptophan degradation via IDO1 through the induction of IFN-γ (Man and Werner-felmayer, 1997).

The vaginal microbiota of a particular CST may affect measurements of kynurenine and tryptophan, as well as the cytokine response. However, because of the complex interactions between the microbial communities in the vaginal tract, host genotype and their immune response, a direct association between CST and IFN-γ levels or kynurenine/tryptophan ratios in specific individuals is difficult to determine and variations may often occur. For example, some women with microbiota of CST I had high kynurenine/tryptophan ratios or high IFN-γ levels. This could be explained by other measurements taken from the same individuals, such as their *Chlamydia* status or the relative abundance of certain bacterial species that may have contributed to these changes. Women with CST I and high vaginal IFN-γ levels, such as 108, 202, 204, and 210, all had low kynurenine/tryptophan ratios, however, they were always accompanied by high IL-17. Participants 210 and 204 had a previous *Chlamydia* infection and participant 202 had a current *Chlamydia* infection. Participant 108 with CST I, was *Chlamydia* negative, however, they had a high relative abundance of indole-producing bacteria (0.23%), and specifically *Propionibacterium acnes*. Participant 106, with CST III and high IFN-γ levels also showed a high relative abundance of *Prevotella* spp. (0.25%). Participant 212, who had CST IV, had no tryptophan or kynurenine in their vaginal secretion sample. This could be due to a low amount of secretions available from this specific individual, which might result in levels that are below the detection of the tryptophan and kynurenine ELISA (tryptophan < 1.2 μg/ml, kynurenine < 47.5 ng/ml). In addition, the participant had presented with *Chlamydia* infection in the previous visit, with CST III and a pH of 5, which supports the observation that vaginal dysbiosis may be associated with *Chlamydia* infection.

Whilst not significant, women with CST IV had higher rates of previous or current chlamydial infection (84%), as well as higher abundance of indole-producing bacteria. The prevalence of indole-producing bacteria within out study cohort was 94%, with the maximum relative abundance of 2.4% of individual's total reads. This suggests that perhaps CST IV provides the most suitable environment for indole production, due to higher pH levels and divers microbial community with low lactic acid production. In addition, the low vaginal tryptophan levels in women with CST IV could be due to tryptophan catabolism into indole by indole-producing bacterial species that contain the *tnaA* gene. This could support the hypothesis that indole-producing bacteria are indeed a contributing factor, to assist chlamydial survival under conditions of immune response-induced tryptophan depletion. This is the first study to analyze the presence of indole producing bacteria in women's vaginal microbiota and their role during *Chlamydia* infections. Previous studies have identified several indole- producing bacteria (Albert et al., 2015), however, we added several more species to our database such as *Propionibacterium acnes*, which were then identified within our cohort.

In this case, the host immune response strategy of killing the *Chlamydia* by starving it from tryptophan might not be as beneficial, (a) Firstly, because *C. trachomatis* genital strains have retained their tryptophan synthase gene and indole-producing bacteria are indeed more prevalent under conditions of vaginal CST IV and (b) secondly, depletion of tryptophan in this environment might compromise host immune defense against opportunistic pathogens and indicate decreased numbers of lactic acid-producing bacterial communities.

In this study, CST IV was found to be associated with the lowest levels of vaginal tryptophan levels, as well as higher abundance of indole-producing bacteria, that can also contribute to (a) elevated pH levels, (b) tryptophan depletion, and (c) supply of exogenous indole that can be used by the *Chlamydia* to survive tryptophan starvation. The most significant risk factor for *C. trachomatis* infection is probably exposure. However, the host microbiome CST present at the time of the exposure might contribute significantly to the effectiveness and type of each individual's immune response to resolve the infection, or not. These factors might also contribute to the level of the *Chlamydia* infection, reproductive outcomes and disorders, and a possibility to a higher susceptibility to opportunistic pathogen colonization during or after chlamydial infections. Perhaps the combination of an active chlamydial infection, along with microbiota of CST IV and especially the presence of certain bacterial species, such as *Streptococcus* and *Peptoniphilus*, might contribute to vaginal dysbiosis and repeated chlamydial infections in the case of repeated exposure.

In the era of “omics” approaches and individualized medicine, our results demonstrate that measuring patient factors, such as vaginal microbiome composition, and metabolic levels (such as tryptophan and kynurenine), in addition to usual diagnosis of sexual contact and STIs, can directly contribute to an individual's sexual health. Other recent research in gut, oral and reproductive-tract sites has shown the importance of the microbiota composition and dysbiosis state on disease processes and on health overall. Our findings suggest that in the case of *C. trachomatis* infections, tryptophan and kynurenine levels, measured directly in vaginal secretions might provide a relatively simple marker for microbiota dysbiosis and even subsequent adverse results from chlamydial infections.

In summary, despite the low number of participants, our pilot study provides preliminary results in which we were able to show for the first time *in vivo*, that high vaginal kynurenine/tryptophan ratio levels correlate with repeated *C. trachomatis* infections and most likely this is due to the immune response inducing IDO1 activity. Tryptophan was significantly lower in women whose microbiota was consistent with CST IV, and there was also a higher relative abundance of indole producers in this type of vaginal microbiota composition, which could explain the low tryptophan levels. High abundance of indole-producing bacteria was found in women PAT, and might indicate a destructive effect of antibiotic treatment on vaginal health after chlamydial infection. Our results shed further light on tryptophan depletion mechanisms during chlamydial infection and indicate the important role of the vaginal microbiota.

## Author contributions

NZ contributed for the project design, methodology preparation, processed the samples, analyzed and interpreted the data and wrote the manuscript. MV conducted the microbiome laboratory work and data analysis. WH contributed to study design, data analysis, writing, and reviewing the manuscript. KT is the sexual health clinic director, and co-ordinated the study and recruitment design, including coordinating the sample collection. PT initiated the study, contributed significantly to the project design, and data interpretation, as well as contributed to the writing and reviewing the manuscript. All authors read and approved the final manuscript.

### Conflict of interest statement

The authors declare that the research was conducted in the absence of any commercial or financial relationships that could be construed as a potential conflict of interest.
