# Supplementary material for: Dysbiosis of the Vaginal Microbiota and Higher Vaginal Kynurenine/Tryptophan Ratio Reveals an Association with Chlamydia trachomatis Genital Infections
Source: Front Cell Infect Microbiol. 2018 Jan 18;8:1. doi: 10.3389/fcimb.2018.00001 (PMC5778109; doi:10.3389/fcimb.2018.00001)
Supplement: Supplementary file 1 [file Table1.DOCX]

Supplementary Table 1: Participants information.
