# Supplementary material for: Dysbiosis of the Vaginal Microbiota and Higher Vaginal Kynurenine/Tryptophan Ratio Reveals an Association with Chlamydia trachomatis Genital Infections
Source: Front Cell Infect Microbiol. 2018 Jan 18;8:1. doi: 10.3389/fcimb.2018.00001 (PMC5778109; doi:10.3389/fcimb.2018.00001)
Supplement: Supplementary file 2 [file SupplementaryFigureS1.pdf]

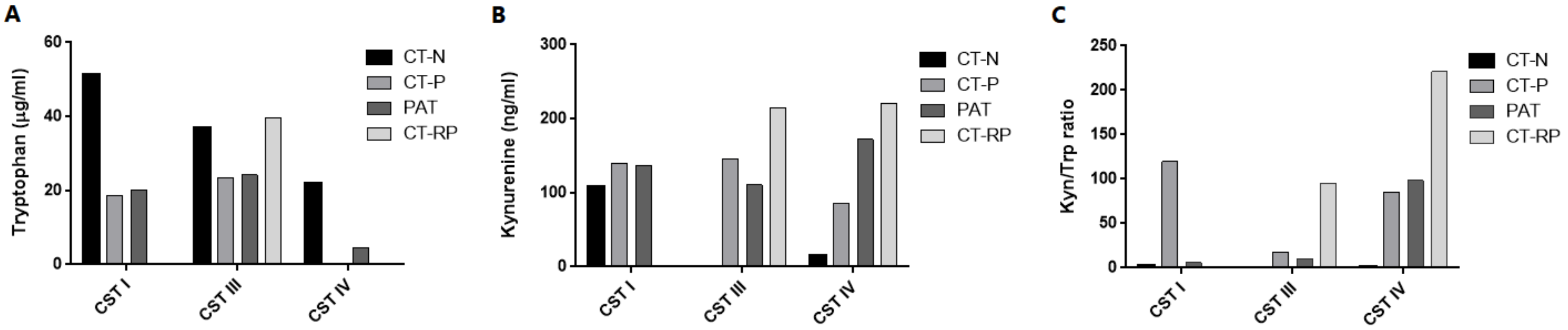

Supplementary Figure S1: Vaginal tryptophan and kynurenine levels according to their CST and *Chlamydia* infections status. A) Tryptophan (µg/ml), B) kynurenine (ng/ml) and C) Kynurenine/tryptophan ratio. The x-axis displays the CST (I, III or IV) of women who were either *Chlamydia* negative (CT-N), *Chlamydia* positive (CT-P), post antibiotic treatment (PAT) or repeated *Chlamydia* infections (CT-RP). Data are presented as mean values.
